# International changes in respiratory syncytial virus (RSV) epidemiology during the COVID‐19 pandemic: Association with school closures

**DOI:** 10.1111/irv.12998

**Published:** 2022-06-22

**Authors:** Marie‐Noëlle Billard, Peter M. van de Ven, Bianca Baraldi, Leyla Kragten‐Tabatabaie, Louis J. Bont, Joanne G. Wildenbeest

**Affiliations:** ^1^ Department of Pediatric Infectious Diseases and Immunology, Wilhelmina Children's Hospital University Medical Center Utrecht Utrecht The Netherlands; ^2^ Department of Data Science and Biostatistics, Julius Center for Health Sciences and Primary Care University Medical Center Utrecht Utrecht The Netherlands; ^3^ University of Udine Udine Italy; ^4^ Julius Clinical Zeist The Netherlands; ^5^ ReSViNET Foundation Zeist The Netherlands

**Keywords:** COVID‐19, non‐pharmaceutical interventions, RSV, SARS‐COV2, seasonality

## Abstract

**Background:**

Little RSV activity was observed during the first expected RSV season since the COVID‐19 pandemic. Multiple countries later experienced out‐of‐season RSV resurgences, yet their association with non‐pharmaceutical interventions (NPIs) is unclear. This study aimed to describe the changes in RSV epidemiology during the COVID‐19 pandemic and to estimate the association between individual NPIs and the RSV resurgences.

**Methods:**

RSV activity from Week (W)12‐2020 to W44‐2021 was compared with three pre‐pandemic seasons using RSV surveillance data from Brazil, Canada, Chile, France, Israel, Japan, South Africa, South Korea, Taiwan, the Netherlands and the United States. Changes in nine NPIs within 10 weeks before RSV resurgences were described. Associations between NPIs and RSV activity were assessed with linear mixed models. Adherence to NPIs was not taken into account.

**Results:**

Average delay of the first RSV season during the COVID‐19 pandemic was 39 weeks (range: 13–88 weeks). Although the delay was <40 weeks in six countries, a missed RSV season was observed in Brazil, Chile, Japan, Canada and South Korea. School closures, workplace closures, and stay‐at‐home requirements were most commonly downgraded before an RSV resurgence. Reopening schools and lifting stay‐at‐home requirements were associated with increases of 1.31% (*p* = 0.04) and 2.27% (*p* = 0.06) in the deviation from expected RSV activity.

**Conclusion:**

The first RSV season during the COVID‐19 pandemic was delayed in the 11 countries included. Reopening of schools was consistently associated with increased RSV activity. As NPIs were often changed concomitantly, the association between RSV activity and school closures may be partly attributed to other NPIs.

## 1 INTRODUCTION

Changes in the seasonality of respiratory viruses including respiratory syncytial virus (RSV) have been a collateral effect of the COVID‐19 pandemic. RSV is highly seasonal and circulates during winter in temperate climates and rainy season in tropical climates.^1^ RSV is a major cause of lower respiratory tract infections in young children and the elderly.^2^, ^3^ In 2015, 3.2 million hospital admissions and 59 000 in‐hospital deaths in children younger than 5 years were attributed to RSV worldwide.^2^

Since the WHO declared COVID‐19 a pandemic in March 2020 (Week (W)11),^4^ unusual RSV circulation patterns have been reported. In both hemispheres, little to no RSV activity was observed during the first expected RSV season, with reductions in RSV detections or acute bronchiolitis hospitalizations up to 90% compared with previous years.^5^, ^6^, ^7^, ^8^ Multiple countries subsequently experienced out‐of‐season increases in RSV cases.^9^, ^10^, ^11^, ^12^ However, the characteristics of RSV resurgences varied greatly. Changes in testing practices, evolution of surveillance systems, or viral interference between RSV and SARS‐COV‐2 may have contributed to these observations.^13^ Also, non‐pharmaceutical interventions (NPIs) that were implemented to slow the spread of COVID‐19 likely played a role.^14^, ^15^

Although several countries have reported their own conclusions regarding the effect of the COVID‐19 pandemic on RSV circulation, few assessed the impact of NPI policies and their timing on RSV activity.^9^, ^10^ In addition, most studies considered lockdown periods, rather than individual NPIs. It is therefore still unclear which NPIs may have triggered RSV resurgences. Understanding the drivers of RSV resurgences may improve forecasting of future epidemics and potentially reveal NPIs that could be implemented annually to reduce RSV circulation.

The two objectives of this study were to describe the international changes in RSV epidemiology during the COVID‐19 pandemic and to improve the understanding of the association between NPIs and RSV resurgences. We hypothesized that including different countries would allow to identify the role of individual measures. First, we described the changes observed in the circulation of RSV using RSV surveillance data from 2017 to 2021 in 11 countries. Then, we identified the NPIs that were downgraded in the 10 weeks before the start of the RSV resurgence in each country. Finally, we investigated the associations between NPIs and the difference between observed and expected RSV activity.

## 2 METHODS

### 2.1 Country selection

**FIGURE 1:**
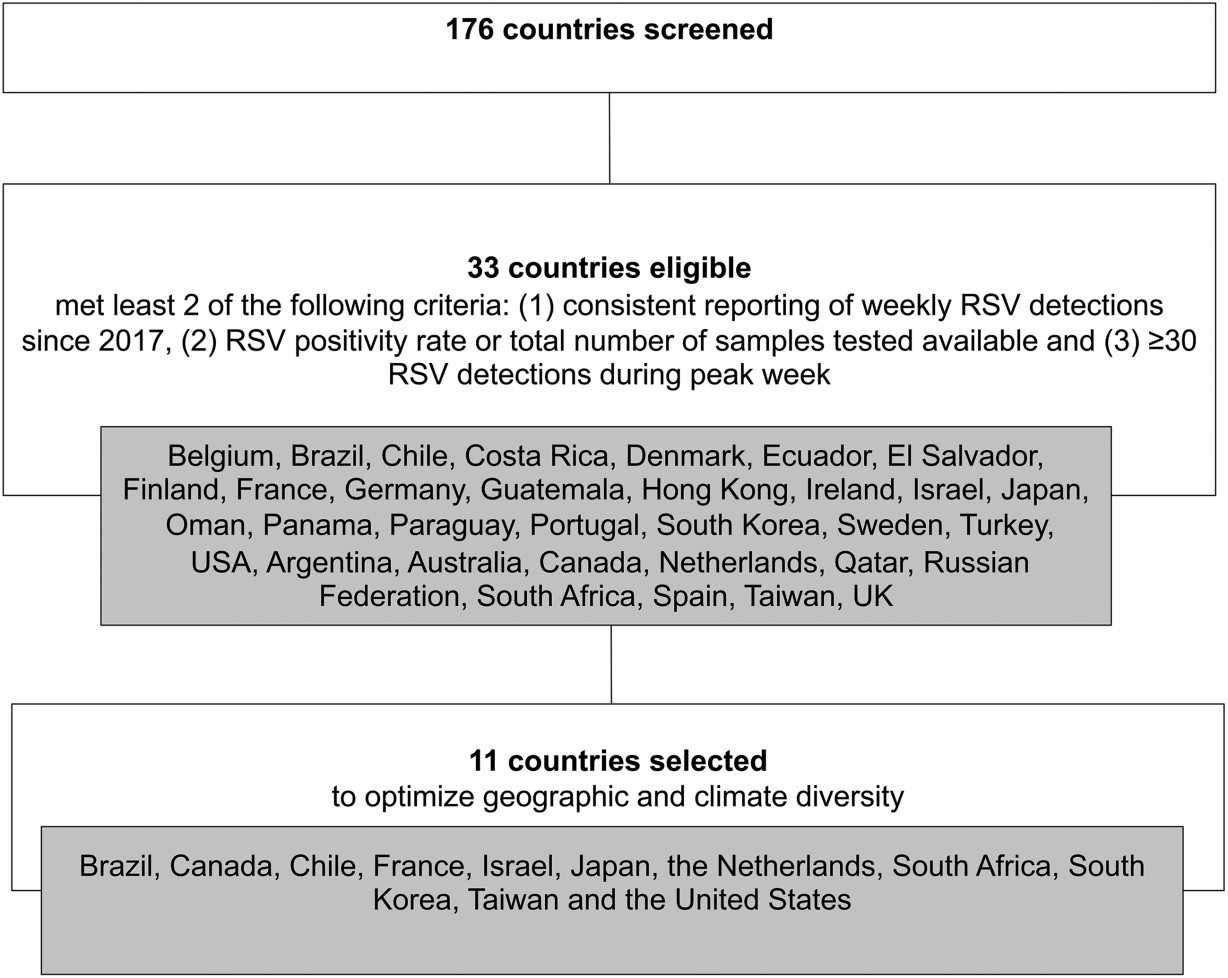
Flowchart of country selection

Countries with publicly available RSV surveillance data were selected between November 2020 and February 2021 (Figure 1). First, we searched three large international infectious diseases surveillance databases to identify countries reporting RSV activity. The ECDC Surveillance Atlas of Infectious Diseases was used as primary source of information for participating EU/EEA countries, PAHO Flunet was used for Pan‐American countries, and the WHO FluNet output was used for all others. Countries that met at least two of the three following criteria were considered eligible: (1) consistent reporting of weekly RSV detections since 2017, (2) RSV positivity rate or total number of samples tested available, and (3) ≥30 RSV detections during peak week. Countries were selected to optimize geographic diversity. In total, 176 countries were screened, 33 were eligible, and 11 were selected: Brazil, Chile, South Africa, Canada, the United States, France, the Netherlands, Israel, Japan, South Korea, and Taiwan. The countries own surveillance outputs were used to collect data (Supplementary Table S1).

### 2.2 RSV surveillance

The RSV surveillance systems varied between the selected countries (Supplementary Table S1). RSV surveillance data were obtained from community clinic networks in South Africa and Israel and from laboratory networks in other countries. One reference laboratory (in Sao Paulo) of the three in Brazil was included. In South Africa, the surveillance had sites in four of nine regions. For the other countries, the entire territory was represented. Data were reported year‐round except in France and Israel that published reports only during winter (W40 to W14). In Israel, reports were published year‐round in 2020. In 2021, another Israeli surveillance system was used between W18 and W40. In France, no data were retrieved from W20‐2020 to W29‐2020 and from W25‐2021 to W29‐2021. We assumed that no RSV was detected in the other weeks. For each week, we extracted the number of RSV detections, number of RSV tests done, and the RSV positivity rate (number of detections divided by the number of tests). In the Netherlands, Japan and Taiwan, the total number of tests was not available and was approximated by the sum of the number of detections for all respiratory viruses.

### 2.3 NPIs

We collected information about individual NPIs from the Oxford COVID‐19 Government Response Tracker (OxCGRT; more information available at https://www.bsg.ox.ac.uk/research/research-projects/covid-19-government-response-tracker). The OxCGRT reports publicly available information on NPIs collected from news articles, government press releases, and briefings. We selected nine NPIs in the OxCGRT: the eight containment and closures policies available, plus facial coverings policies. We did not include COVID‐19 specific or economic measures that were outside the scope of this study. The nine NPIs were school closures, workplace closures, facial covering policies, gathering restrictions, cancellation of public events, stay‐at‐home restrictions, public transport closures, restriction on internal movement in the country (e.g., within regions or states), and international travel controls (Supplementary Table S2). The NPIs stringency levels (0 to 2, 3, or 4, with 0 indicating no measures) were grouped when necessary to avoid empty categories. From the OxCGRT, we also extracted the stringency index, which is a composite score of the eight containment and closure policies and the public information campaign variable (scale ranging from 0 to 100). The OxCGRT reports information daily. For this study, the highest stringency level of the week for each country was used.

### 2.4 Outcomes

To describe the changes in RSV epidemiology, we reported the delay of the onset and the peak of the first RSV epidemic observed in each country during the COVID‐19 pandemic, relative to the expected onset and peak of the RSV season. We qualitatively assessed the meteorological season in which onset of that first RSV epidemic was expected and observed. We described changes in the shape of the RSV peaks during the COVID‐19 pandemic relative to previous RSV seasons. Then, we examined which NPIs were downgraded before the RSV resurgences. To identify NPIs that could explain the deviation in RSV cases from pre‐pandemic RSV epidemics, we assessed associations between the weekly difference between observed and expected RSV activity in the pandemic period W18–‐2020 to W44‐2021 and the concurrent stringency index and level of NPIs.

### 2.5 Statistical analysis

We determined the onset and offset week of RSV seasons for each country using the average annual percentage (AAP) method.^1^, ^16^ The pre‐pandemic period consisted of years 2017–2019 (W01–W52) for southern hemisphere countries plus Taiwan and Japan and years 2016–2017 to 2018–2019 (W27–W26) for other countries. For each week in the pre‐pandemic period, the AAP was calculated as the number of RSV detections divided by the total number of RSV detections in the year. The weekly AAPs were sorted, and the minimal number of weeks required to include 75% of the year's RSV detections was labeled “epidemic weeks.” The RSV season was defined as the longest streak of consecutive epidemic weeks, with 2‐week gaps accepted. An average AAP score was calculated for each week of the year across the pre‐pandemic seasons. The expected onset for the first RSV season during the COVID‐19 pandemic was determined as the week in 2020 that corresponded to the onset week based on the average pre‐pandemic AAP scores. The first observed RSV epidemic was determined using the AAP method if a peak was observed visually and the number of cases had returned to baseline by the end of the observation period. Otherwise, the onset was defined as the first week with >10% RSV detections. The peak week was the week with the highest number of cases detected in the year.

The delay in onset was calculated as the total number of weeks between the expected and the observed onset of the first RSV epidemic during the COVID‐19 pandemic. Delays in onset <40 weeks (i.e., 9 months) were classified as “delayed season,” whereas longer delays were classified as “missed season.”

To describe the NPIs that were downgraded before the RSV resurgence, the start of the RSV increase was defined as the first of the consecutive weeks with >0.5% of RSV detections that included the first RSV epidemic during the COVID‐19 pandemic, with 2‐week gaps accepted. The RSV detection rate of 0.5% was selected based on the observation that RSV detection rates were lower while RSV was not circulating. We arbitrarily selected a period of 10 weeks before the start of the increase in RSV to allow enough time for detection by the selected surveillance system despite differences in surveillance population coverage, country size, and population density. We reported the number of weeks between downgrading the NPIs and the peak of the RSV epidemic.

Association between NPIs and deviations in RSV activity compared with pre‐pandemic seasons were tested using linear mixed models (LMM). As the dependent variable, we considered the weekly deviation in RSV detection rate, defined as the difference between the observed weekly RSV detection rates and the average pre‐pandemic detection rates for the same week number. Random effects for hemisphere and country were used with countries nested within hemisphere. The stringency index or the individual NPIs were included as fixed effects. NPIs were entered as continuous or categorical according to best fit, defined as the model with smallest Akaike information criterion (AIC). First, each NPI was included in a separate model (referred to as “single‐NPI models”) with hemisphere and country included as random effect. All NPIs that showed an association with *p* < 0.5 were included in the full model from which the final model was selected with a backward elimination procedure. At each step, we removed the single NPI whose omission yielded the best fit model (smallest AIC) among all candidate models with a single NPI removed. This was repeated until the model contained only NPIs that were associated with average deviance in RSV activity (*p* < 0.1) and whose contribution to the model was significant (ANOVA for nested models, *p* < 0.1) or whose removal worsened model fit when assessed using AIC. As some levels of NPIs were only rarely observed, we performed a sensitivity analysis where NPIs were dichotomized. A cutoff value was chosen independently for each country and based on the frequency distribution of weeks over the categories and univariate analyses where level of the NPI was entered as a categorical variable (Supplementary Table S3).

LMM were fitted in RStudio Version 1.3 with the maximum likelihood method and included a first‐order autoregressive error (“lme” function from R package “nlme”).

### 2.6 Ethics

As this study used only publicly accessible surveillance data, it did not require ethics approval.

## 3 RESULTS

### 3.1 Pre‐pandemic RSV seasonality

During the pre‐pandemic years, one peak of RSV was observed annually, except in Taiwan where two peaks occurred in 2017 and 2018. As expected, the RSV season onset and offset weeks varied between countries but stayed consistent over the years in most countries (Supplementary Table S3). Larger variability was observed in Taiwan, Brazil, and South Africa where the annual number of RSV detections was generally lower and the RSV season was longer than in the other countries (19–26 weeks vs. 10–14 weeks). In the (sub)tropical countries (latitude of −23° to −34° and 23° to 34°), pre‐pandemic RSV seasons started in summer, corresponding to the rainy or humid period in South Africa, Brazil, and Taiwan. Pre‐pandemic RSV season onset occurred during the winter season in most temperate countries (latitude ≥35° or ≤−35°), which included Chile, France, The Netherlands, Israel, the United States, and Canada. However, Japan's recent pre‐pandemic RSV seasons started at the end of summer, and South Korea seasons started in autumn.

### 3.2 Pandemic impact on onset of RSV season

**FIGURE 2:**
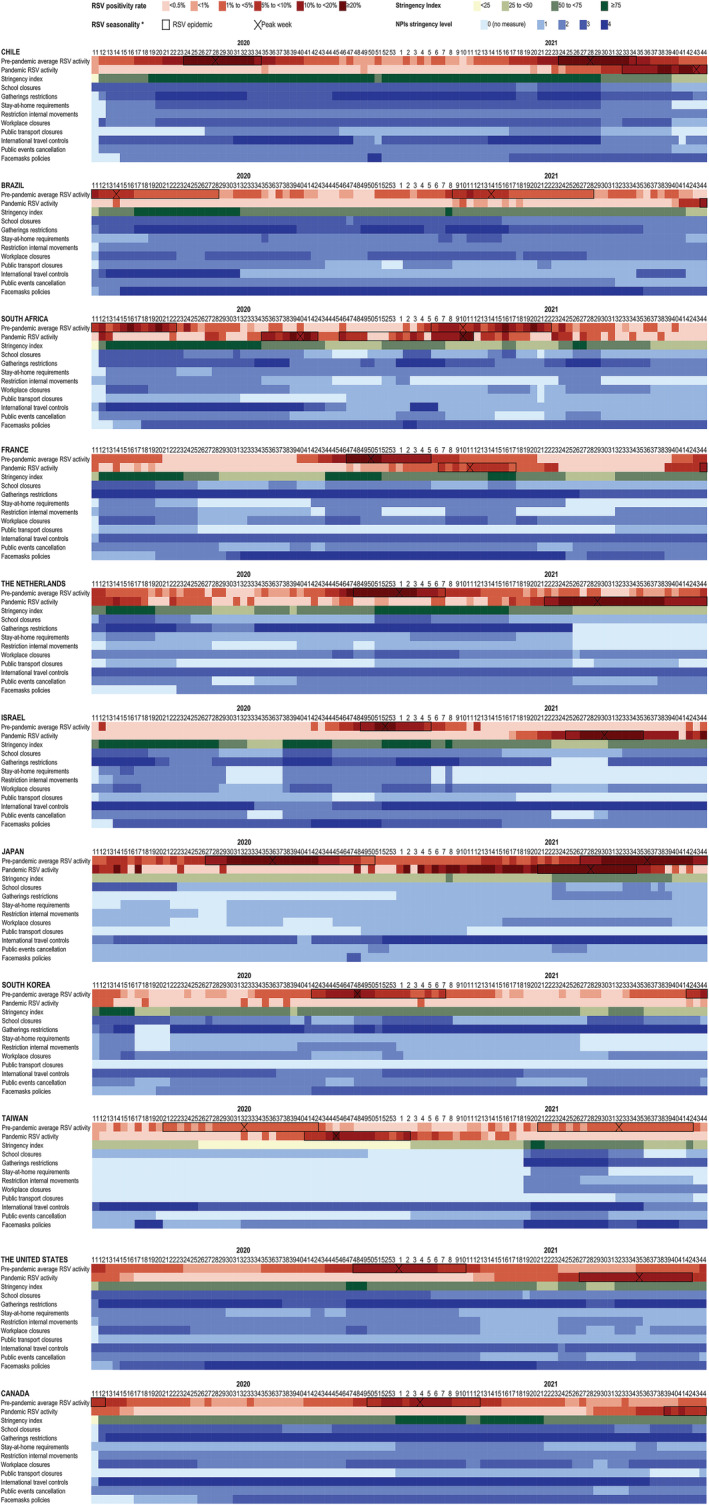
Changes in non‐pharmaceutical interventions, weekly proportions of RSV detections and RSV epidemics from W11–2020 to W44–2021 and in pre‐pandemic seasons, in 11 countries

COVID‐19 was declared a pandemic in March 2020 (W11‐2020). The 2019–2020 RSV season ended in the eight preceding weeks in Canada, France, Israel, the Netherlands, South Korea, and the United States (Table 1). However, only in Israel the number of detections had returned to baseline by W11‐2020 (Figure 2). In the southern hemisphere, the number of RSV detections was increasing in South Africa and Brazil. The season was expected to start later in Chile. Japan and Taiwan were also between RSV seasons.

**TABLE 1:** Characteristics of the pre‐pandemic RSV season and first pandemic RSV season, changes in RSV epidemiology, per country

	South Africa	Brazil	Chile	France	The Netherlands	Israel	The United States	Canada	Japan	Taiwan	South Korea
First RSV epidemic during COVID‐19 pandemic											
Expected epidemic period											
Onset week	W06‐2020	W09‐2020	W24‐2020	W47‐2020	W48‐2020	W49‐2020	W48‐2020	W50‐2020	W27‐2020	W21‐2020	W42‐2020
Peak week	W10‐2020	W14‐2020	W34‐2020	W50‐2020	W01‐2021	W52‐2020	W01‐2021	W04‐2021	W36‐2020	W32‐2020	W48‐2020
Offset week	W22‐2020	W28‐2020	W28‐2020	W05‐2021	W07‐2021	W05‐2021	W10‐2021	W12‐2021	W50‐2020	W42‐2020	W07‐2021
Observed epidemic period											
Onset week	W35‐2020	W44‐2021	W33‐2021	W07‐2021	W22‐2021	W25‐2021	W27‐2021	W39‐2021	W21‐2021	W41‐2020	‐
Peak week	W40‐2020	‐	W43‐2021	W11‐2021	W29‐2021	W30‐2021	W35‐2021	‐	W28‐2021	W45‐2020	‐
Offset week	W42‐2020	‐	‐	W17‐2021	W44‐2021	W35‐2021	W42‐2021	‐	W34‐2021	W02‐2020	‐
Second RSV epidemic period during COVID‐10 pandemic											
Onset week	W46‐2020		‐	W44‐2021	‐	‐	‐	‐	‐	‐	‐
Peak week	W10‐2021	‐	‐	‐	‐	‐	‐	‐	‐	‐	‐
Offset week	W11‐2021	‐	‐	‐	‐	‐	‐	‐	‐	‐	‐
Delay of first RSV epidemic during COVID‐19 pandemic											
Delayed/missed season	Delayed	Missed	Missed	Delayed	Delayed	Delayed	Delayed	Missed	Missed	Delayed	Missed
Onset delay (weeks)	29	88	62	13	27	29	32	42	47	20	‐
Peak delay (weeks)	30	‐	‐	14	28	31	34	‐	45	13	‐
Season of RSV epidemic onset^a^											
Pre‐pandemic RSV seasons	Summer	Summer	Winter	Autumn	Winter	Winter	Winter	Winter	Summer	Summer	Autumn
First RSV epidemic during COVID‐19 pandemic	Winter	Winter	Winter	Winter	Summer	Summer	Summer	Autumn	Spring	Winter	‐

^a^ The meteorological seasons were defined as follows. For France, the Netherlands, the United States, Canada, Japan, and South Korea: spring from March to May, summer from June to August, autumn from September to November and winter from December to February. For South Africa and Chile: spring from September to November, summer from December to February, autumn from March to May and winter from June to August. For Israel and Taiwan: summer from May to September and winter from October to April. For Brazil: summer from October to April, winter from May to September.

During the pandemic, a delay in onset of the first RSV epidemic during the COVID‐19 pandemic was observed in all 11 countries (Table 1). Compared with what was expected based on pre‐pandemic seasons, the delay in onset was 39 weeks on average and ranged from 13 weeks in France to 88 weeks in Brazil. The delay in onset was less than 40 weeks in South Africa, France, the Netherlands, Israel, the United States, and Taiwan. Brazil, Canada, Chile, Japan, and South Korea were classified as having missed a season. No RSV resurgence has been detected in South Korea up to W44‐2021 (>49 weeks delay).

Delayed seasons were generally characterized by a peak outside the normal period of high RSV activity (Table 1). The first RSV epidemic during the COVID‐19 pandemic started in the opposite season (e.g., summer instead of winter) in South Africa, the Netherlands, Israel, and the United States. However, in France, the first RSV epidemic during the COVID‐19 pandemic started 13 weeks late in winter instead of late autumn. In most of the countries that missed a season, the onset of the first RSV epidemic during the COVID‐19 pandemic occurred the following year during or close to the expected meteorological season for high RSV activity: in winter in Chile, late in the spring instead of summer in Japan and in autumn instead of winter in Canada.

### 3.3 Impact on shape of RSV epidemic

Whereas in South Africa pre‐pandemic RSV seasons showed one peak, two consecutive periods of high RSV activity were observed in 2020–2021 (Figure 2). After a first peak when RSV activity was expected to be low (2020‐W35 to 2020‐W42), RSV positivity rate decreased. Then, a second peak of similar height and overlapping with the normal period of high RSV activity was observed (2020‐W46 to 2021‐W11). In total, RSV circulated for 30 weeks while pre‐pandemic seasons on average lasted 17 weeks. In Brazil, a small increase of RSV detections was observed when RSV activity was expected to be high (W09‐2021 to W18‐2021), but weekly proportion of RSV detection stayed below 5%. The 10% of weekly RSV detection was reached for the first time in W44‐2021, 88 weeks later than expected based on pre‐pandemic seasons.

On the contrary, pre‐pandemic RSV seasons in Taiwan could have two annual peaks, but only one was observed during the COVID‐19 pandemic (Figure 2). The RSV positivity rate at the peak was higher than in pre‐pandemic seasons. However, the positivity rate may have been overestimated as the total number of RSV tests was approximated using respiratory viruses' detections.

Interestingly, France was the only country where a second and separate period of high RSV activity was observed during the COVID‐19 pandemic with over 10% RSV detections in W44‐2021 (Figure 2).

### 3.4 NPIs before RSV resurgence

In the 10 weeks preceding the RSV increase, the most commonly downgraded NPIs were school closures, workplace closures, and stay‐at‐home requirements, respectively, in six, four, and four countries (Table 2). No country downgraded public transport closures during that period. The number of downgraded NPIs varied from one to six between countries. Multiple NPIs were generally downgraded at the same time. School closures, workplace closures, and stay‐at‐home requirements were downgraded on average 21 and 22 weeks before the peak of RSV detections. However, there were large variations between countries.

**TABLE 2:** Non‐pharmaceutical interventions that were downgraded in the 10 weeks preceding the start of the RSV resurgence (for week >0.5% RSV detections) and number of weeks between changes in NPIs and the peak of the first RSV epidemic during the COVID‐19 pandemic

	South Africa	Brazil	Chile	France	The Netherlands	Israel	The United States	Canada	Japan	Taiwan	South Korea
Start of the RSV increase	W27‐2020	W40‐2021	W20‐2021	W47‐2020	W11‐2021	W17‐2021	W12‐2021	W28‐2021	W01‐2021	W32‐2020	‐
Peak week first RSV epidemic during COVID‐19	W40‐2020	‐	W43‐2021	W11‐2021	W29‐2021	W30‐2021	W35‐2021	‐	W28‐2021	W45‐2020	‐
Time from downgrading NPIs to RSV peak (weeks)											
School closures	16		25	23	23	14	29				
Workplace closures	21				19	21					
Stay‐at‐home requirements	17					21	25				
Gatherings restrictions			25			20					
Public events cancellation				21					28		
Facemasks policy						14			32		
Public transport closures											
Restriction internal movements				21		21					
International travel controls										19	

### 3.5 Association between NPIs and RSV resurgences

We hypothesized that lower NPIs levels were associated with higher difference between observed and expected RSV activity. As expected, the stringency index was negatively and significantly associated with the difference in RSV activity (Table 3). A 10‐point decrease in the stringency index was associated with an average absolute increase in the difference between observed and expected RSV positivity rate of 0.8%.

**TABLE 3:** Association between non‐pharmaceutical interventions stringency levels and changes in RSV activity during the pandemic, defined as the difference between expected and observed proportions of weekly RSV detections, in linear mixed models, including hemisphere and country as random effects

	Univariate models		Full model		Final model		Proportion of variance explained^a^
	Coeff	*p*‐value	Coeff	*p*‐value	Coeff	*p*‐value	
**Model 1: Stringency index**							
Stringency index (per 1 point)	**−0.08**	**0.04**	‐	‐	‐	‐	**1.2%**
**Model 2: NPIs after grouping**							
School closures	**−1.57**	**0.01**	**−1.09**	**0.10**	**−1.31**	**0.04**	**1.4%**
Stay‐at‐home requirements	**−2.86**	**0.01**	−1.69	0.19	**−2.27**	**0.06**	**1.7%**
Restriction internal movements	**−1.33**	**0.04**	−0.64	0.38	‐	‐	‐
Public transport closures	**−1.73**	**0.06**	−1.07	0.25	‐	‐	‐
Gatherings restrictions	−1.10	0.20	−0.21	0.83	‐	‐	‐
Public events cancellation	−0.70	0.47	0.26	0.81	‐	‐	‐
Workplace closures	−0.27	0.68	‐	‐	‐	‐	‐
Facemasks policy	0.07	0.99	‐	‐	‐	‐	‐
International travel controls	0.003	0.99	‐	‐	‐	‐	‐
**Sensitivity analysis: NPIs dichotomized**							
School closures	**−2.04**	**0.02**	−1.64	0.20	**−1.94**	**0.03**	**0.7%**
Stay‐at‐home requirements	−1.07	0.29	−0.50	0.07	‐	‐	‐
Restriction internal movements	**−2.31**	**0.03**	−1.45	0.63	‐	‐	**‐**
Public transport closures	**−1.92**	**0.06**	−1.50	0.21	**−1.77**	**0.08**	**0.6%**
Gatherings restrictions	−0.38	0.70	‐	‐	‐	‐	‐
Public events cancellation	−0.87	0.38	−0.18	0.86	‐	‐	‐
Workplace closures	−0.10	0.91	‐	‐	‐	‐	‐
Facemasks policy	0.04	0.98	‐	‐	‐	‐	‐
International travel controls	0.35	0.79	‐	‐	‐	‐	‐

^a^ R^2^ calculated with Nakagawa and Schielzeth approach.

*Note*: Values in bold refer to variable that were statistically significant (p < 0.1) as per the methods.

In the single‐NPI models, school closures (−1.57, *p* = 0.01), stay‐at‐home requirements (−2.86, *p* = 0.01), restrictions of internal movements (−1.33, *p* = 0.04), and public transport closures (−1.73, *p* = 0.06) were all found to be negatively associated with the difference between observed and expected RSV positivity rate. Only school closure and stay‐at‐home requirement contributed to the model and were included in the final model. Reopening schools and lifting stay‐at‐home requirement were associated with an absolute increase of 1.31% (*p* = 0.04) and 2.27% (*p* = 0.06) in the deviation from expected RSV activity. In the sensitivity analysis, reopening schools was associated with an absolute increase of 1.94% (*p* = 0.03) in the in the deviation from expected RSV activity. Whereas stay‐at‐home requirement was not included in the final model when dichotomized, reopening public transport was associated with an absolute increase of 1.77% (*p* = 0.08) in the deviation from expected RSV activity as a binary variable. Overall, the proportion of the variance explained by the NPI was small (<2%). No evidence of multicollinearity was detected the models (variance inflation factor <2).

## 4 DISCUSSION

This study aimed to describe and compare changes in RSV seasonality observed during the COVID‐19 pandemic in 11 countries and to explore their association with NPI policies using publicly accessible surveillance data. In all countries included in the analysis, the first RSV epidemic during the COVID pandemic was delayed (13–88 weeks, mean: 39 weeks). Whereas one RSV season was missed in Brazil, Chile, Japan, Canada, and South Korea (delay ≥40 weeks), the onset of the first RSV season during the COVID pandemic was <40 weeks late in the other six countries. RSV activity was consistently associated with school closures in the descriptive analysis and the models.

School closures were the NPI most often downgraded before the RSV resurgences and lower levels of school closures were systematically associated with increased RSV activity in univariate and multivariate analyses. This association was expected as children are recognized as the main transmission group for RSV.^17^ However, school closures were not downgraded in all countries before the RSV resurgence. Accordingly, whereas some previous publications also underlined the role of school closures, others found no temporal link with RSV resurgences.^9^, ^11^, ^18^, ^19^ Lower levels of school closures are probably not sufficient to trigger RSV resurgences.

Other NPIs may have contributed to RSV resurgences. Similarly to the effect of school closures, lower levels of stay‐at‐home requirements would likely increase between‐households social interactions in all age groups, and its association with RSV activity was expected. Surprisingly, public transport closure was found to be associated with RSV activity when dichotomized. Although not often implemented, the periods when public transport were closed corresponded with high levels of NPIs stringency in general. Conversely, interstate travels has been suggested as potential facilitating factor in Australia.^7^, ^9^ Although lower levels of internal travel were associated in RSV activity in the single NPI model, it was not included in the final model. Overall, our analysis point toward a strong effect of school closures but probably did not sufficiently disentangled the effects of other NPIs.

Other factors than NPIs could contribute to the differences observed between countries. Meteorological factors have been found to be associated with RSV seasonality.^20^ For RSV, the effect of population immunity seems more important. During the COVID‐19 pandemic, RSV circulation was limited, and the susceptible fraction of the population increased through births and waning immunity, as RSV infections do not provide long‐lasting protection.^21^, ^22^ Early models predicted the out‐of‐season resurgences that have been observed due to build‐up in susceptible individuals.^23^ Viral interference with other respiratory viruses may also play a role. In the few countries that reported both viruses' activity since the start of the COVID‐19 pandemic, rhinovirus peaked before the RSV resurgence.^24^, ^25^, ^26^, ^27^ Negative interactions between RSV and rhinovirus have been previously reported^28^, ^29^ and may have contributed to delaying RSV resurgences after downgrading NPIs.

In addition to their impact on the circulation of respiratory viruses, negative socioeconomic impact related to the large‐scale implementation of NPIs have been observed.^30^ Consequently, implementing mandatory social distancing each winter to prevent the circulation of respiratory viruses is not realistic. However, models predicting future RSV seasons assumed an overall effect of NPIs on RSV transmission, whereas NPIs target different social groups.^23^ According to these models, the magnitude of the changes in RSV seasonality is expected to increase with the duration of NPIs implementation.^23^ As NPIs are still largely implemented, it is important to better understand their associations with RSV activity in order to accurately predict coming RSV seasons.

The main strengths of this study are its international perspective and the analysis of NPIs individually. This study also has some important limitations. First, we extracted RSV data from various surveillance systems. To limit the impact of changes in the number of tests done, we defined RSV seasons annually and used weekly proportion of detections. All pre‐pandemic RSV seasons fell within the expected period of high RSV activity for the country. The AAP method may result in shorter epidemic periods than other methods, which could have resulted in an overestimation of the delay in onset of the first RSV epidemic during the COVID‐19 pandemic.^16^ Although RSV reporting was inconsistent in Brazil in 2020, there is no indication of a period of high RSV activity.^31^ In Israel, RSV activity was retrieved from another surveillance system from W18‐2021 to W40‐2021. As this period covered the entire RSV epidemic, the determination of the epidemic period was likely not impacted. NPI data were extracted from a unique global tracker that was not built for RSV. For example, daycare closures were considered part of the more general workplace closures. Furthermore, the NPIs were at country level and did not account for within‐country variations. In addition, NPI policies do not necessarily translate to behavior. Adherence may differ between countries and change over time through pandemic fatigue. As NPIs were only implemented in pandemic period, we had to use the deviation between the observed and expected weekly RSV detection rate to infer on the potential impact of NPIs on RSV activity. The deviation took on negative or positive values, when RSV activity was, respectively, lower or higher than expected based on the pre‐pandemic average in the same week. The coefficients therefore reflect the contribution of NPI to preventing RSV circulation and to RSV resurgences. However, school closures remained the main predictor when we modeled weekly RSV detection rates (data not shown). Finally, we did not take into account other possible drivers of RSV activity. In particular, meteorological factors may be correlated with RSV activity and to NPIs stringency level via COVID‐19 transmission. Not accounting for meteorological factors could have biased the models estimates. However, in a recent study that included meteorological factors, the risk of RSV rebound in the 10 weeks that followed full reopening of schools was significantly increased (hazard ratio = 23 [95% CI: 1–496]).^32^

Overall, we described the patterns of the first RSV epidemic during the COVID‐19 pandemic in 11 countries. We identified that lower levels of school closures and possibly stay‐at‐home requirements were associated with increased RSV activity. It is important to accurately predict RSV seasons, especially with the continuing COVID‐19 pandemic and novel variants arising. To better estimate the effect of each NPI, future studies should include other known drivers of RSV seasonality and other respiratory viruses' activity and explore associations in additional countries.

## AUTHOR CONTRIBUTIONS

**Marie‐Noëlle Billard:** Data curation; formal analysis. **Peter van de Ven:** Formal analysis; supervision. **Bianca Baraldi:** Data curation; formal analysis. **Leyla Kragten‐Tabatabaie:** Conceptualization; funding acquisition. **Louis Bont:** Conceptualization; funding acquisition. **Joanne Wildenbeest:** Conceptualization; supervision.

### PEER REVIEW

The peer review history for this article is available at https://publons.com/publon/10.1111/irv.12998.

## Supporting information


**Supplementary Table S1:** Characteristics of RSV surveillance in the 11 selected countries and source of RSV surveillance data
**Supplementary Table S2** **:** Codebook of non‐pharmaceutical interventions.
**Supplementary Table S3:** RSV season onset week, offset week peak weeks and duration of the season in weeks.

## Data Availability

The data supporting the findings of this study were derived from publicly accessible resources. The list and links to these resources are available in Supplementary Table S1.
