# Supplementary material for: International changes in respiratory syncytial virus (RSV) epidemiology during the COVID‐19 pandemic: Association with school closures
Source: Influenza Other Respir Viruses. 2022 Jun 22;16(5):926–36. doi: 10.1111/irv.12998 (PMC9343326; doi:10.1111/irv.12998)
Supplement: Supplementary file 1 — Supplementary Table S1: Characteristics of RSV surveillance in the 11 selected countries and source of RSV surveillance data Supplementary Table S2 : Codebook of non‐pharmaceutical interventions. Supplementary Table S3: RSV season onset week, offset week peak weeks and duration of the season in weeks. [file IRV-16-926-s001.docx]

**Supplementary Material**

Contents

[Supplementary table S1: Characteristics of RSV surveillance in the 11 selected countries and source of RSV surveillance data 2](#_Toc94002064)

[Supplementary table S2: Codebook of non-pharmaceutical interventions. 3](#_Toc94002065)

[Supplementary Table S3: RSV season onset week, offset week peak weeks and duration of the season in weeks. 4](#_Toc94002066)

## **Supplementary table S1: Characteristics of RSV surveillance in the 11 selected countries and source of RSV surveillance data**

| **Country** | **Name of the surveillance system** | **Surveillance type** | **Samples origin** | **Geographic representativity** | **Time of functionement** | **Unavailable indicator** | **Source RSV** | |
| --- | --- | --- | --- | --- | --- | --- | --- | --- |
| Brazil | PAHO Influenza and Other Respiratory Viruses surveillance in the Americas. | sentinel | laboratories | 1 of 3 reference laboratories, in Sao Paulo | year-round | - | [Link](http://ais.paho.org/phip/viz/ed_flu.asp) | |
| Canada | The Respiratory Virus Detection Surveillance System | sentinel | laboratories | entire country | year-round | - | [Link](https://www.canada.ca/en/public-health/services/surveillance/respiratory-virus-detections-canada.html) | |
| Chile | The Influenza and other respiratory viruses laboratory surveillance | sentinel | laboratories | entire country | year-round | - | [Link](https://www.ispch.cl/virusrespiratorios/) | |
| France | National Network of Hospital Laboratories (RENAL) | non-sentinel | laboratories | entire country | Winter season: from W40 to W14 (all year in 2020) | - | [Link](https://www.santepubliquefrance.fr/maladies-et-traumatismes/maladies-et-infections-respiratoires/bronchiolitehttp:/atlas.ecdc.europa.eu/public/index.aspx) | |
| Israel | Israel Center for Disease Control Ministry of Health | sentinel | community clinic network | entire country | Winter season: from W40 to W14 (all year in 2020) | - | [Link](https://www.health.gov.il/UnitsOffice/ICDC/Infectious_diseases/Flu/Pages/FWR.aspx) | |
| Japan | The infectious agent surveillance in Japan | sentinel | laboratories | entire country | year-round | Total number of RSV tests. The sum of detections for all respiratory viruses was used as proxy* | [Link](https://www.niid.go.jp/niid/en/iasr/510-surveillance/iasr/graphs/2295-iasrgv4e.html) | |
| South Africa | The systematic Influenza-Like-Illness surveillance program | sentinel | community clinic network | 3 (<2020) and 4 (since 2020) of the 9 provinces | year-round | - | [Link](https://www.nicd.ac.za/diseases-a-z-index/disease-index-covid-19/surveillance-reports/weekly-respiratory-pathogens-surveillance-report-week/) | |
| South Korea | The Korea Influenza and Respiratory Viruses Surveillance System (KINRESS) | sentinel | laboratories | entire country | year-round | - | [Link](http://www.kdca.go.kr/board/board.es?mid=a30502000000&bid=0032) | |
| Taiwan | The national laboratory surveillance network | non sentinel | laboratories | entire country | year-round | Total number of RSV tests. The sum of detections for all respiratory viruses was used as proxy** | [Link](https://nidss.cdc.gov.tw/en/Home/Index?op=9) | |
| The Netherlands | The National Institute for Public Health and the Environment(RIVM) | sentinel | laboratories | entire country | year-round | Total number of RSV tests. The sum of detections for all respiratory viruses was used as proxy¶ | [Link](https://www.rivm.nl/virologische-weekstaten) | |
| The United States | The National Respiratory and Enteric Virus Surveillance System (NREVSS) | sentinel | laboratories | entire country | year-round | - | [Link](https://www.cdc.gov/surveillance/nrevss/rsv/index.html) | |
| * RSV, Rhinovirus, Adenovirus, Human Metapneumovirus, Parainfluenza virus and Influenza virus. ** RSV, Parainfluenza, HSV, CMV, Adenovirus, Influenza virus ¶ RSV, Influenza (A, B and C), Adenoviruses, Coronaviruses, Enteroviruses, hMPV, Parainfluenza (1, 2, 3, 4), Parechoviruses and Rhinoviruses | | | | | | | |  |

## **Supplementary table S2: Codebook of non-pharmaceutical interventions.**

| **Non-pharmaceutical interventions** | **Original coding** | **After grouping categories** | **After dichotomization** |
| --- | --- | --- | --- |
| **school closure** | 0 no measures | 0 no measures | 0-2; 3 for Brazil, Canada Chile, The United States |
|  | 1 recommended closing | 1 recommended closing |  |
|  | 2 required at some level | 2 required at some level | 0-1, 2-3 for other countries |
|  | 3 required closings | 3 required closings |  |
| **workplace closure** | 0 no measures | 0 no measures | 0-2, 3 for Brazil, Canada, Chile, France, the Netherlands, Israel |
|  | 1 recommended closing | 1 recommended closing |  |
|  | 2 required at some level | 2 required at some level | 0-1, 2-3 for other countries |
|  | 3 required closings | 3 required closings |  |
| **facial covering policies** | 0 No measures | 0, 1, 2 partial requirement | 0-3, 4 for Brazil, France, South Africa, United States |
|  | 1 Recommended |  |  |
|  | 2 Required in some specified shared/public spaces |  | 0-2, 2-4 for Canada, Chile, Israel, Japan, Taiwan |
|  | 3 Required in all shared/public spaces | 3 required in all shared/public spaces |  |
|  | 4 Required outside the home at all times | 4 required outside the home at all times | 0-1,2-4 for other countries |
| **gathering restrictions** | 0 no restrictions | 0, 1 no restriction or only on very large gatherings (>1,00) | 0-3, 4 for Brazil, Canada, Chile, France, Israel, the Netherlands, the United States |
|  | 1 restrictions on very large gatherings over 1,000 |  |  |
|  | 2 restrictions on 101-1,000 | 2, 3 restrictions on 11-1,000 | 0-2, 3-4 for South Africa |
|  | 3 restrictions on 11-100 |  |  |
|  | 4 restrictions on 10 or fewer | 4 restrictions on 10 or fewer | 0-1, 2-4 for other countries |
| **cancellation of public events** | 0 no measures | 0, 1 no or recommended cancellation | 0-1, 2 for Brazil, Canada, Chile, France, Israel, Japan, the Netherlands, South Africa, the United States |
|  | 1 recommended cancellation |  |  |
|  | 2 required cancellation | 2 required cancellation | 0, 1-2 for other countries |
| **stay at home restriction** | 0 no measures | 0 no measures | 0-2,3 for Brazil, Chile |
|  | 1 recommended | 1, 2 recommended or partially required | 0-1, 2-3 for Canada, France, Israel, the Netherlands, South Africa, Taiwan, the United States |
|  | 2 required with exceptions |  |  |
|  | 3 required with minimal exceptions | 3 required with minimal exceptions | 0, 1-3 for other countries |
| **public transport closures** | 0 no measures | 0 no measures | 0-1, 2 in Brazil, Chile |
|  | 1 recommended closings | 1 recommended closings |  |
|  | 2 required closings | 2 required closings | 0,1-2 in other countries |
| **restrictions on internal movement (within country)** | 0 no measures | 0 no measures | 0-1, 2 for Brazil, Canada, Chile, France, Israel, the United States, South Africa |
|  | 1 recommended not to travel between regions/cities | 1 recommended not to travel between regions/cities |  |
|  | 2 restricted internal movement | 2 restricted internal movement | 0,1-2 for other countries |
| **restrictions on international travel** | 0 no restrictions | 0, 1, 2 no restrictions, screening or quarantine upon arrival | 0-3, 4 for Brazil, Canada, France, Israel, Japan, the Netherlands, Taiwan |
|  | 1 screening arrivals |  |  |
|  | 2 quarantine arrivals |  |  |
|  | 3 ban arrivals from some regions | 3 ban arrivals from some regions | 0-2, 3 for other countries |
|  | 4 ban arrivals from all regions or total border closure | 4 ban arrivals from all regions or total border closure |  |

## **Supplementary Table S3: RSV season onset week, offset week peak weeks and duration of the season in weeks.**

| **Country** | **Season** | **Peak week** | **RSV season (Onset- Offset)** | |
| --- | --- | --- | --- | --- |
| Brazil | 2017 | W14 | W05-W21 | |
|  | 2018 | W14 | W11-W23 | |
|  | 2019 | W11 | W01-W22 | |
| South Africa | 2017 | W10 | W06-W17 | |
|  | 2018 | W15 | W06-W19 | |
|  | 2019 | W19 | W11-W28 | |
| Chile | 2017 | W28 | W23-W33 | |
|  | 2018 | W28 | W23-W33 | |
|  | 2019 | W28 | W24-W34 | |
| Taiwan | 2017 | W39 | W12-W22 & W27-W44 | |
|  | 2018 | W36 | W19-W23 & W31-W43 | |
|  | 2019 | W33 | W26-W38 | |
| The United States | 2016-2017 | W01 | W50-W10 | |
|  | 2017-2018 | W04 | W48-W10 | |
|  | 2018-2019 | W01 | W48-W11 | |
|  | 2019-2020* | W01 | W47-W08 | |
| The Netherlands | 2016-2017 | W52 | W47-W04 | |
|  | 2017-2018 | W01 | W49-W08 | |
|  | 2018-2019 | W01 | W49-W07 | |
|  | 2019-2020* | W51 | W49-W08 | |
| Israel | 2016-2017 | W52 | W48-W05 | |
|  | 2017-2018 | W01 | W46-W06 | |
|  | 2018-2019 | W51 | W49-W05 | |
|  | 2019-2020* | W51 | W47-W03 | |
| France | 2016-2017 | W51 | W48-W06 | |
|  | 2017-2018 | W51 | W46-W05 | |
|  | 2018-2019 | W01 | W48-W06 | |
|  | 2019-2020* | W51 | W48-W06 | |
| Japan | 2016-2017 | W41 | W30-W04 | |
|  | 2017-2018 | W35 | W27-W51 | |
|  | 2018-2019 | W36 | W27-W48 | |
|  | 2019-2020* | W36 | W27-W46 | |
| South Korea | 2016-2017 | W49 | W42-W06 | |
|  | 2017-2018 | W49 | W39-W02 | |
|  | 2018-2019 | W48 | W43-W07 | |
|  | 2019-2020* | W51,5** | W45-W06 | |
| Canada | 2016-2017 | W04 | W50-W10 | |
|  | 2017-2018 | W07 | W51-W14 | |
|  | 2018-2019 | W04 | W51-W12 | |
|  | 2019-2020* | W06 | W51-W11 | |
| * 2019-2020 was not used to estimate pre-pandemic average  ** equal number of RSV samples in W51 and W52. | | | |  |
